# Supplementary material for: Molecular and Pathogenic Characterization of Fusarium Species Associated with Corm Rot Disease in Saffron from China
Source: J Fungi (Basel). 2022 May 17;8(5):515. doi: 10.3390/jof8050515 (PMC9147734; doi:10.3390/jof8050515)
Supplement: Supplementary file 1 [file jof-08-00515-s001.zip › jof-1708845-supplementary.pdf]

Journal of *Fungi*

MDPI

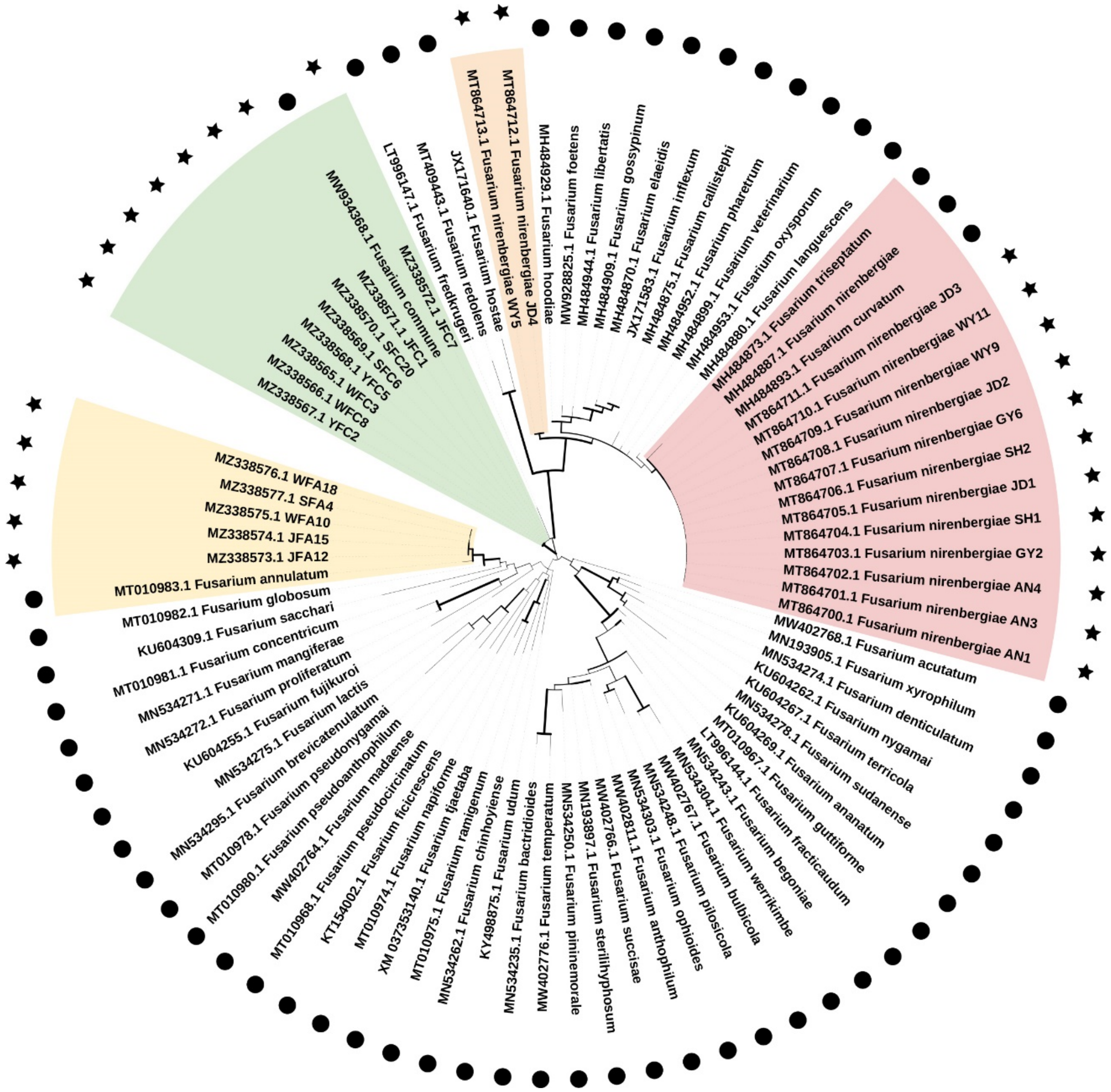

Figure S1. Phylogeny of *Fusarium* isolates from saffron, based on the partial *rpb2* gene. Sequences from type strains are indicated by a circle (●). Sequences from strains isolated from saffron are indicated by a star (★). Clades including saffron isolates are shaded in color. Evolutionary history was inferred by using the Maximum Likelihood method and Tamura-Nei model [45]. The tree with the highest log likelihood (-3483.33) is shown. Initial tree(s) for the heuristic search were obtained automatically by applying Neighbor-Join and BioNJ algorithms to a matrix of pairwise distances estimated using the Tamura-Nei model, and then selecting the topology with superior log likelihood value. The tree is drawn to scale, with branch lengths measured in the number of substitutions per site. This analysis involved 84 nucleotide sequences. All positions with less than 95% site coverage were eliminated, i.e., fewer than 5% alignment gaps, missing data, and ambiguous bases were allowed at any position (partial deletion option). There were a total of 648 positions in the final dataset. Evolutionary analyses were conducted in MEGA X version 10.2.4 [25, 46] and the tree image was generated using the Interactive Tree Of Life (iTOL) v5 [47].

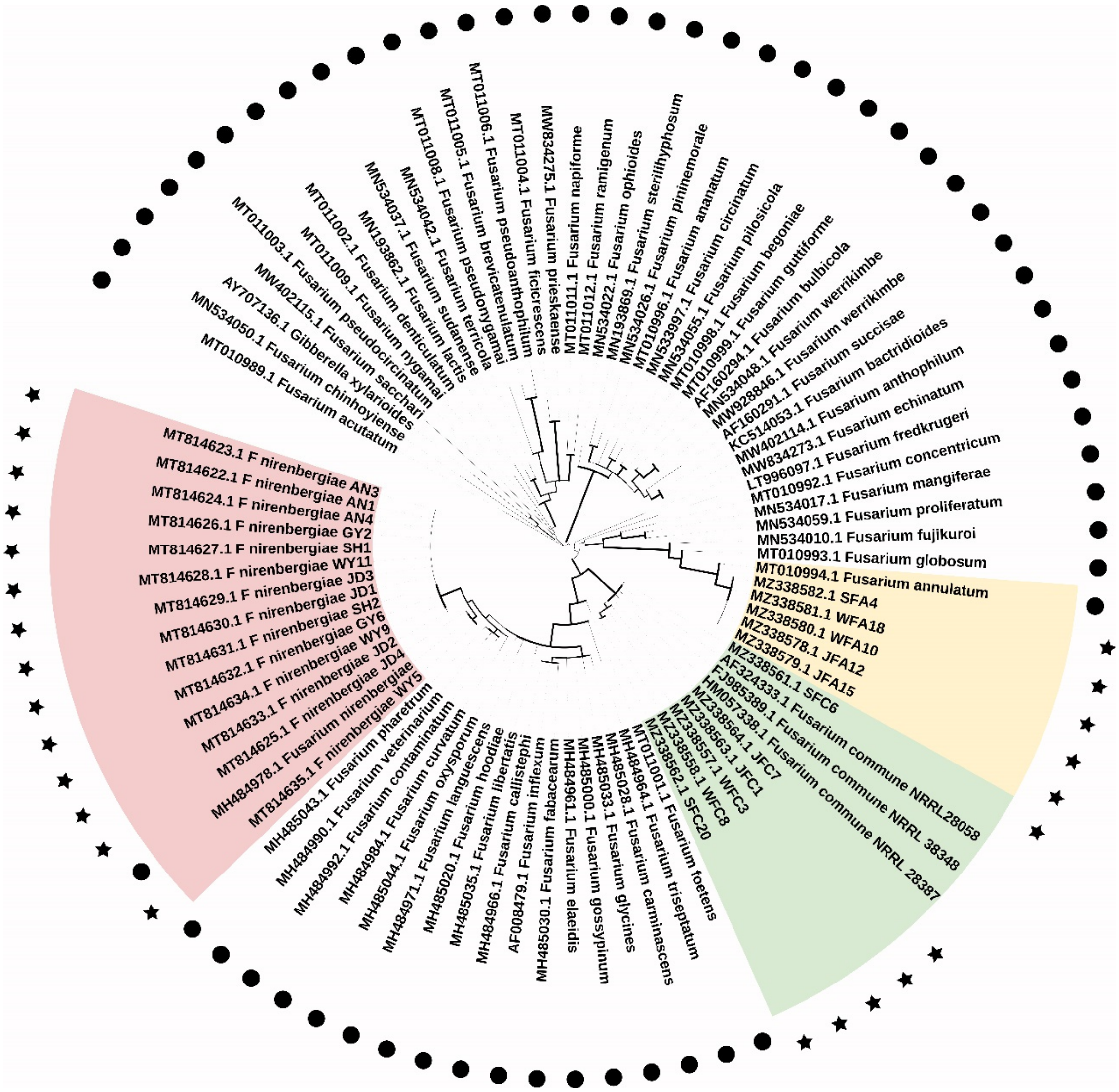

Figure S2. Phylogeny of *Fusarium* isolates from saffron, based on the partial *tef1* gene. Sequences from type strains are indicated by a circle (●). Sequences from strains isolated from saffron are indicated by a star (★). Clades including saffron isolates are shaded in color. Evolutionary history was inferred by using the Maximum Likelihood method and Tamura-Nei model [45]. The tree with the highest log likelihood (-3460.04) is shown. The percentage of trees in which the associated taxa clustered together is shown next to the branches. Initial tree(s) for the heuristic search were obtained automatically by applying Neighbor-Join and BioNJ algorithms to a matrix of pairwise distances estimated using the Tamura-Nei model, and then selecting the topology with superior log likelihood value. The tree is drawn to scale, with branch lengths measured in the number of substitutions per site. This analysis involved 87 nucleotide sequences. All positions with less than 95% site coverage were eliminated, i.e., fewer than 5% alignment gaps, missing data, and ambiguous bases were allowed at any position (partial deletion option). There were a total of 583 positions in the final dataset. Evolutionary analyses were conducted in MEGA X version 10.2.4 [25, 46] and the tree image was generated using the Interactive Tree Of Life (iTOL) v5 [47].
